# Response to Limited surface impacts of the January 2021 sudden stratospheric warming

**DOI:** 10.1038/s41467-023-38772-3

**Published:** 2023-06-07

**Authors:** Judah Cohen, Laurie Agel, Mathew Barlow, Chaim I. Garfinkel, Ian White

**Affiliations:** 1 Atmospheric and Environmental Research Inc., Lexington, MA 02421 USA; 2 Department of Civil and Environmental Engineering, Massachusetts Institute of Technology, Cambridge, MA 02139 USA; 3 Department of Environmental, Earth, and Atmospheric Sciences, University of Massachusetts Lowell, Lowell, MA USA; 4 The Hebrew University of Jerusalem, Institute of Earth Sciences, Edmond J. Safra Campus, Jerusalem, Israel; 5 Department of Geosciences, Faculty of Exact Sciences, Tel Aviv University, Tel Aviv, Israel

**Keywords:** Atmospheric dynamics, Climate and Earth system modelling

**arising from** N. A. Davis et al. *Nature Communications* 10.1038/s41467-022-28836-1 (2022)

Cohen et al.^1^ argue that Rossby wave energy reflection in the stratosphere contributed to the historic United States (US) cold air outbreak of February 2021. In their later article, Davis et al.^2^ present global climate model (GCM) sensitivity experiments where they conclude that the disruptions of the polar vortex and related reflection of planetary wave activity in the stratosphere during January and February had no discernible contribution to the February 2021 North American, including the US, cold air outbreak^1,3^. Though we acknowledge the importance of these and similar experiments, we also note concerns regarding the methodology in the experiments as well as the ability of the model to reproduce the February 2021 US cold wave. This debate has important implications for the accurate prediction of extreme weather and the application of numerical models to predict severe winter weather related to polar vortex variability at longer leads.

## Diagnosing wave reflection and implications for methodology

**Fig. 1:**
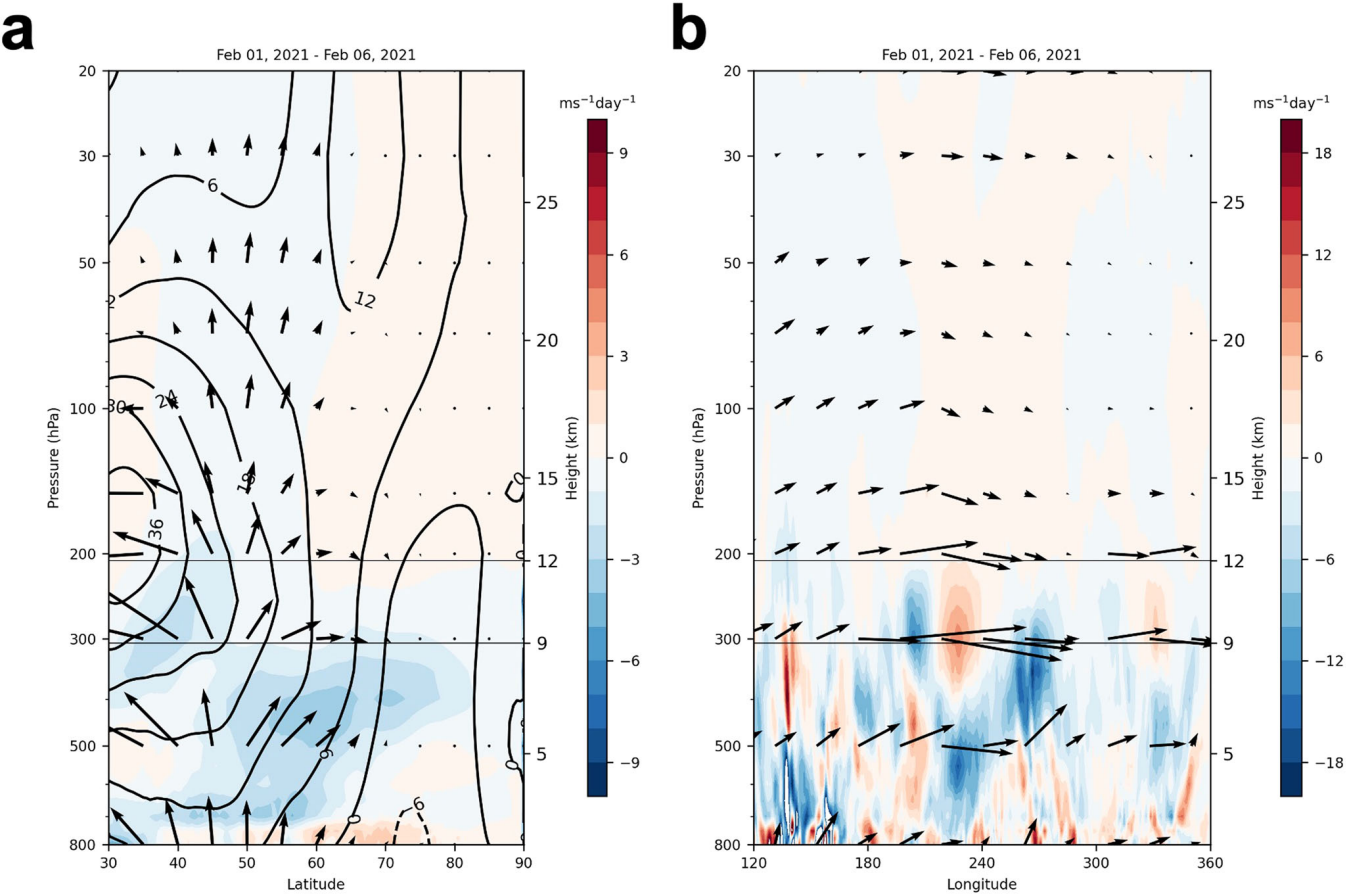
Wave energy convergence critical for North American cold wave occurs near the tropopause. **a** Wave activity flux (WAF; vectors), zonal mean zonal wind (contours), and divergence of wave activity flux (shading) for 1–6 February 2021 for **a** zonal mean WAF or Eliassen-Palm flux in latitude–height cross-section and **b** WAF averaged between 40 and 75°N in longitude–height cross-section. The atmospheric layer between 9 and 12 km is delineated by solid black lines. WAF computed from MERRA2 data^11^ using the scaling of Jucker^12^ as in Davis et al.^2^.

Davis et al.^2^ argue that downward reflected energy from the stratosphere did not contribute to the US cold wave of February 2021. We agree with Davis et al.^2^ that the source of the energy originated in the troposphere and not the stratosphere. We argue that, as shown in Figs. 5 and 7 from Davis et al.^2^ and in Fig. 1a, at least some of the energy that contributed to the amplification of the tropospheric North American wave pattern originated over Northern Asia, mostly between 90–150°E and 45–60°N but then was refelccted downward off the lower stratospheric polar vortex back towards the troposphere.

The wave activity flux (WAF) vectors as shown in Figs. 6 and 7 of Davis et al.^2^ and Fig. 1 are downward in the stratosphere but upward in the troposphere over North America. This is consistent with previous studies of reflected/stretched polar vortex events that show upward WAF in the troposphere over North America (Kodera et al.^4^ Fig. 3; Matthias and Kretschmer^5^ Figs. 4 and 5) and not downward as assumed by Davis et al.^2^. This is likely important as it facilitates convergence of WAF near the tropopause, which is crucial in amplifying the North American standing wave supportive of cold air outbreaks. As noted by Davis et al.^2^, WAF passing through a region is not sufficient to amplify or dampen a wave pattern, rather a convergence or divergence of WAF is required. Incorrect simulation of this convergence and/or divergence would most likely result in a model prediction that is too mild compared to the observations, as obtained in Davis et al.^2^ and discussed in the next subsection.

As seen in Figs. 6 and 7 from Davis et al.^2^ and Fig. 1, convergence of WAF over western North America is observed centered around the tropopause in the upper troposphere and lower stratosphere but is missing in all of the model forecasts. We agree with Davis et al.^2,6^ that WAF convergence between 180°W and 90°W is important for wave amplification over North America. However, the assertion that convergence to the east in the stratosphere (east of 90°W shown in Fig. 7 of Davis et al.^2^) is not important is not supported and contradicts a previous study that argued it contributes to wave amplification and cold air outbreaks across North America^7^. During wave reflection, planetary waves tilt eastward with height over North America so the tropospheric low pressure trough (roughly between 120°W and 90°W) that delivers the cold to the US is connected to the stratospheric low pressure trough (between 90°W and 30°W) which is the region where WAF convergence occurs in the stratosphere (Fig. 7 of Davis et al.^2^).

Furthermore, Davis et al.^2^ initialized the model with reanalysis data fully at 9 km and then nudge to a different state tapering off up to 12 km in their scrambled stratospheric initial conditions (ICs) experiments. Due to the varying degree of nudging, the model atmosphere is constrained by the observations either fully or partially throughout this layer at initialization of the experiment (but not in the forecasts); in Fig. 1a we have delineated this layer with dashed lines, which includes the critical region for wave reflection near the tropopause and lower stratosphere.

The methodological approach of Davis et al.^2,6^ treats the polar vortex and associated wave reflection as occurring in the mid to upper stratosphere. However, the polar vortex extends throughout the stratosphere starting at the tropopause^8^. Moreover, there is no physical reason to expect reflection to occur only in the upper stratosphere. In fact, Matthias and Kretschmer^4^ defined their reflective index based on eddy poleward heat flux at 100 hPa in the lower stratosphere. Furthermore, a recent study found that the static stability near the tropopause and in the lowermost stratosphere is sufficient to initiate wave reflection^9^, which is in the region of the initialization in the troposphere only (scrambled stratosphere ICs) experiments performed by Davis et al.^2^. Therefore, we argue that the methodological choice of transitioning between observed and scrambled conditions in the 9–12 km layer cannot fully remove the influence of the lower stratosphere.

Davis et al.^6^ provides new analysis arguing that the contribution from above the lower stratosphere was trivial to the critical WAF convergence resulting in the amplified wave pattern across North America during the period of February 1–6, 2021. From our own WAF diagnostics preceding the US cold wave of February 12–18, 2021^3^, a more detailed analysis is required from January 26 through February 12, 2021 to better understand the role of WAF propagation and WAF convergence in the troposphere and stratosphere. But regardless, the new analysis presented by Davis et al.^6^ prima facie corroborates a previous study that demonstrated that the influence of wave reflection in the stratosphere is to trap wave numbers 2 and 3 in the lower stratosphere and troposphere. The suppressed vertical wave propagation then contributes to North Pacific blocking in the troposphere crucial for North American cold waves^7^.

## Cold in the United States and model fidelity

**Fig. 2:**
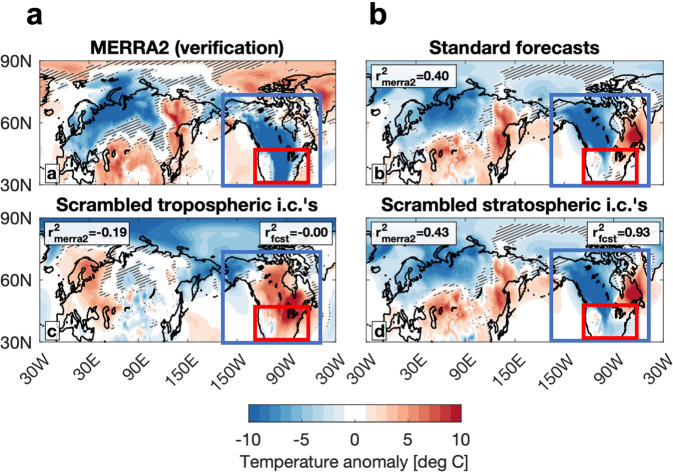
Model forecasts simulate North American cold due to internal variability but not due to forced variability. Surface temperature anomalies from **a** MERRA2 and **b** ensemble-mean surface temperature anomalies in the period February 8–12, 2021 as shown in Fig. S3 in Davis et al.^2^. We included a blue box to delineate North America and a red box to delineate the United States. The predicted North American cold wave is limited to Canada.

Davis et al.^2,6^ interpret their model forecasts initialized on February 1 and 8, both with the standard forecast and the scrambled forecasts, as predicting a cold air outbreak comparable to the coldest event in the MERRA2 reanalysis. The ability of their model to simulate the observed event is shown in their Fig. S3 in Davis et al.^2^ and Fig. 3 in Davis et al.^6^. We repeat their figure here in our Fig. 2, but also include a box for both North America (blue) and the US (red). Their original analysis exclusively uses a North American average. Though we agree that the model forecasts accurately predicted a severe cold air outbreak for western Canada, there are two critical differences from the observed cold air outbreak. First, the model forecasts also predicted a notable warm surface temperature anomaly in eastern Canada that is absent in the observations, which suggests there may be substantial differences from observations in the tropospheric circulation. More importantly, however, the model predictions do not reproduce the severe cold observed over the US, the region of interest in Cohen et al.^3^. There is a small area of slightly colder-than-average temperatures in the central US but also some warmer-than-average areas as well. Thus, the North American average that their analysis focuses on, and that their conclusions are drawn from, is representative only of western Canada. That one ensemble member can predict cold approaching the magnitude of the observed anomaly^6^ suggests that the model can predict the cold attributable to natural variability but not the cold that is attributable to external forcings such as wave reflection.

It appears, based on Figs. 5 and 7 in Davis et al.^2^, that the model did not simulate the wave reflection as observed, likely contributing to the model not reproducing the extremity of the cold across the US. This could be the explanation for the discrepancy between model temperature forecasts and observations.

## Alternative modeling approaches

In summary, we question the applicability of the Davis et al.^2^ modeling experiments to assessing the role of lower stratospheric wave reflection in the February 2021 cold wave, especially over the US. As a way forward, we propose three modeling experiments (one of which was included in Davis et al.^2^), with the initial conditions based on observations as in Davis et al.^2^, but better suited to diagnose the involvement of wave reflection in this event. (1) To more cleanly assess the role of the troposphere, the upper bounds of the “scrambled stratosphere” runs should be changed from 9 km (full nudging)/12 km (no nudging) to something lower in altitude, for example 4 km (full nudging)/7 km (no nudging). This run would be considered the new “scrambled tropopause and stratosphere”. (2) To assess the role of the mid-stratosphere and upper stratosphere, Davis et al.’s^2^ “scrambled troposphere” can be retained as is; however it should be reconsidered as the “scrambled troposphere and lower stratosphere”, as the nudging in the initialization is full only until 20 km (and not at all below 15 km), while the polar winter tropopause is usually much lower. (3) To assess the role of the tropopause region and lower stratosphere, a possible third experiment in which the troposphere is scrambled (full scrambling below ~4 km, transitioning to observations by ~7 km) and also the mid- and upper- stratosphere is scrambled (full scrambling above 20 km transitioning to observations by ~15 km) may elucidate the role of the region near the tropopause for wave reflection. By combining these three experiments, perhaps further light could be shed on which regions matter most for the surface impacts. Furthermore, the underestimation of the extent of the surface temperature response in North America, and especially the lack of a response over the United States, may be ameliorated if nudging was applied over the duration of the forecast and not just at the initialization. Nudging experiments are planned as part of the Stratospheric Nudging And Predictable Surface Impacts^10^ experiments and are likely to advance our understanding of the role of the polar vortex on the weather. It would be worth systematically exploring which regions need to be nudged (starting from the entire tropopause region and stratosphere, and gradually removing the nudging), for future work. An examination of the circulation differences associated with the model’s failure to extend the severe cold into the US would also improve our understanding of the tropospheric dynamics of the event.

Davis et al.^2,6^ have performed interesting modeling experiments that question the role of the stratosphere and the polar vortex on the North American cold wave of 2021. While we agree that modeling experiments are important to testing the role of the stratosphere in this event, the difficulties of the model in reproducing the observed structure of wave reflection and convergence in the control run, as well as the relationship of the nudging approach to the lower stratosphere, raise concerns for us over the interpretation of the model results. Davis et al.^6^ also present additional observational diagnostics showing that upward WAF in the troposphere and the lower stratosphere is the primary contributor to WAF convergence in that region; we agree with this result but question the assertion that convergence in the stratosphere to the east played no role. In fact, the observational analysis of WAF propagation and convergence presented in Davis et al.^2,6^ provides strong empirical underpinnings for the theoretical construct on how wave reflection in the stratosphere contributes to North American cold waves^7^. We suggest that additional modeling experiments and observational analysis are necessary to determine the causal role of the lower stratosphere in determining the occurrence of this convergence.

## Data Availability

No new data was created for this manuscript.

## Code availability

No new code was written for this manuscript.
